# Oncofertility in patients with stage I epithelial ovarian cancer: fertility-sparing surgery in young women of reproductive age

**DOI:** 10.1186/s12957-017-1222-4

**Published:** 2017-08-15

**Authors:** Xuan Jiang, Jiaxin Yang, Mei Yu, Weimin Xie, Dongyan Cao, Ming Wu, Lingya Pan, Huifang Huang, Yan You, Keng Shen

**Affiliations:** 1 Department of Obstetrics and Gynecology, Peking Union Medical College Hospital, Chinese Academy of Medical Science & Peking Union Medical College, Shuai Fu Yuan No.1, Dongcheng District, Beijing, 100730 People’s Republic of China; 2 Department of Pathology, Peking Union Medical College Hospital, Chinese Academy of Medical Science & Peking Union Medical College, Beijing, People’s Republic of China

**Keywords:** Fertility-sparing surgery, Epithelial ovarian cancer, Stage I, Reproductive age, Survival

## Abstract

**Background:**

Fertility-sparing surgery is indicated for patients with stage I epithelial ovarian cancers. We sought to evaluate the clinical outcomes and oncofertility in a cohort of patients of reproductive age with stage I epithelial ovarian cancer (EOC).

**Methods:**

Overall, 108 patients of reproductive age (≤ 40 years) diagnosed with stage I EOC who were treated at Peking Union Medical College Hospital between 1999 and 2013 were included in the study. The Kaplan-Meier model and Cox regression analyses were used for the survival analysis.

**Results:**

The type of surgery included fertility-sparing surgery (FSS) (48.1%) and radical surgery (RS) (51.9%). After a median follow-up of 83 months, we observed that grade 3 or clear-cell carcinoma was the only independent risk factor for disease-free survival and tumor-specific survival in the multivariate analysis. Patients with grade 3 or clear-cell carcinoma tended to be older than 30 years, have endometriosis, and undergo RS (*p* < 0.05). Fertility-sparing surgery did not affect disease-free survival or tumor-specific survival among patients of reproductive age with stage I EOC and among high-risk patients with stage IC2-3, grade 3, or clear-cell carcinoma. Thirty-four out of 52 (65.4%) FSS patients attempted to get pregnant. Twenty-eight (82.4%) achieved a successful pregnancy with a full-term delivery.

**Conclusions:**

Grade 3 or clear-cell carcinoma was the only independent risk factor for survival of patients of reproductive age with stage I EOC. FSS can be safely performed on patients of reproductive age with grade 1-2, stage I EOC. The safety of FSS for grade 3 and clear-cell carcinoma warrants further investigation.

## Background

Epithelial ovarian cancer (EOC) is the most lethal of malignant ovarian tumors [1]. Clinicians have been reluctant to perform fertility-sparing surgery in certain groups of patients with stage I epithelial ovarian cancers, including those with poorly differentiated tumors or clear-cell carcinomas. Based on current evidence-based medicine, fertility-sparing surgery is indicated for patients with stage I epithelial ovarian cancers [2, 3]. Fertility-sparing surgery is only meaningful in patients with type I epithelial ovarian cancer, including endometrioid, mucinous, low-grade serous, and clear-cell carcinomas [4]. Patients with high-grade serous ovarian cancers can relapse within a short period of time and are not advised to keep the contralateral ovary [5].

Preserving the high survival rate for stage I EOC is of utmost importance. There has been increasing interest in fertility-sparing surgery that does not negatively affect survival for these patients. The reason for keeping the contralateral ovary and uterus is that most of these patients can survive for a long time and could potentially die from other conditions instead of tumor recurrence. Most studies have suggested that oncologic and reproductive outcomes of patients who undergo FSS are favorable [6, 7].

Approximately, 30% of patients with epithelial ovarian cancers are diagnosed with stage I and 13% are younger than 40 years [8]. As the child-bearing age is becoming increasingly delayed, some patients may be diagnosed with malignant ovarian tumors before child-bearing. Radical surgery can be unacceptable in these patients, because they still wish to conceive.

In this study, we compared the survival of women of reproductive age with newly diagnosed stage I EOC who underwent FSS, with those who underwent RS. We also performed a subgroup analysis of patients with high-risk disease, including grade 3, clear-cell, or stage IC2-3 tumors, and assessed the pattern of recurrence between groups, as well as subsequent reproductive outcomes of women undergoing FSS.

## Methods

The study protocol was approved by the ethics committees of Peking Union Medical College Hospital. We retrospectively identified patients with stage I EOC aged ≤ 40 years at diagnosis, who underwent primary staging surgery at the Department of Obstetrics and Gynecology of Peking Union Medical College Hospital (PUMCH) between 1999 and 2013. The diagnoses and staging were reassessed based on the fourth edition of the World Health Organization Classification of Tumors of Female Reproductive Organs and International Federation of Gynecology and Obstetrics (FIGO) 2014 staging system. According to FIGO 2014, stages IC1, IC2, and IC3 were defined as intraoperative spillage, preoperative capsule rupture or surface invasion, and positive cytology results, respectively.

Patients were eligible for inclusion if they were surgically and pathologically diagnosed with stage I (i.e., IA-B, IC1, IC2, IC3) EOC (i.e., mucinous, serous, endometrioid, and clear-cell carcinoma (CCC)) and aged ≤ 40 years at diagnosis. Patients with rare or special histological type of epithelial carcinoma (malignant Brenner tumors, squamous cell carcinomas, undifferentiated carcinomas, and epithelial carcinomas complicated with sarcoma components), carcinoma in situ, or borderline ovarian tumor were excluded from the study. Patients with incomplete clinical and pathological or follow-up information and those with disease extending beyond stage I and undergoing FSS were also excluded.

FSS was recommended to young patients with FIGO stage IA or IC1, grade 1-2 tumor, and non-clear-cell histology, with a strong desire to stay fertile and who could be monitored during followed-ups at a gynecologic oncology outpatient clinic. RS was suggested for those patients with high-risk disease, including grade 3, clear-cell histology, or stage IC2-3 tumors, based on intraoperative or final paraffin pathological diagnoses.

Comprehensive staging surgery included omentectomy, retroperitoneal, and/or para-aortic lymphadenectomy, appendectomy, excision of all suspicious nodes, peritoneal washings, and multiple-site random peritoneal biopsies. Fertility-sparing surgery included ipsilateral adnexectomy and biopsy or wedge excision of contralateral ovary. Radical surgery included hysterectomy and bilateral adnexectomy. Two independent pathologists with extensive experience in gynecological pathology reviewed all of the pathological slides and were blinded to patient outcomes.

Within the study period, adjuvant chemotherapy was administered to patients considered at increased risk for recurrence (FIGO stage IC1 or more, grade 2-3 tumor, or clear-cell histology) after the primary surgery. Chemotherapy regimens consisted of TC (paclitaxel and carboplatin), TP (paclitaxel and cisplatin), PC (cyclophosphamide and cisplatin), CC (cyclophosphamide and carboplatin), or PAC (cisplatin, epirubicin, and cyclophosphamide). The majority of patients received TC and PC chemotherapy. The number of cycles ranged from three to nine after tailoring therapy to patients.

After completion of the initial treatment, patients were followed-up monthly for the first 6 months, every 2 months after 6 months, every 3 months after 1 year, every 6 months after 2 years, and every year after 5 years. Clinical examinations performed at each visit included pelvic examination, ultrasonography scan, evaluation of carbohydrate antigen 125 (CA 125), and other previously elevated serum tumor markers. Patients were contacted by telephone or letter to obtain regular follow-up information when it was not available.

Recurrence was documented using histologic evidence of disease via tumor biopsy, fine-needle biopsy, or the appearance of new lesions on imaging examination. Disease-free survival (DFS) was defined as the time interval from the date of primary surgery to the date of disease recurrence or censoring during the last follow-up. Tumor-specific survival (TSS) was defined as the time interval from the date of the primary surgery to the date of death or censoring during the last follow-up. Endometriosis-associated ovarian cancer (EAOC) was defined as the coexistence of cancer and endometriosis in the same or contralateral ovary or extra-ovarian endometriosis [9]. All EAOCs were confirmed pathologically. We evaluated four categories of pretreatment serum tumor markers: CA 125, CA 19-9, carcinoembryonic antigen (CEA), and alpha fetoprotein (AFP). The normal upper limits of serum tumor markers CA 125, CA 19-9, CEA, and AFP were 35 and 37 U/mL and 5 and 20 ng/mL, respectively. Patients were considered to have an elevated serum tumor marker when any of these serum tumor markers were elevated.

### Statistical analyses

Comparisons between the FSS and RS groups were performed using an independent *t* test, the Mann-Whitney *U* test, or the chi-square test as appropriate. DFS and TSS times were estimated using the Kaplan-Meier model and compared between the groups using the log-rank test. The Cox regression model was used for multivariate analysis. Variables with *p* < 0.1 in the univariate analyses were included in the multivariate analyses. Hazard ratios (HR) and 95% confidence intervals (CI) were calculated for the significant variable in the multivariate analyses. All *p* values reported were two tailed; *p* values < 0.05 were considered statistically significant. We performed statistical analyses using IBM SPSS 22.0 software for Macintosh and Graph Pad Prism 5.0.

## Results

### Study population

**Fig. 1:**
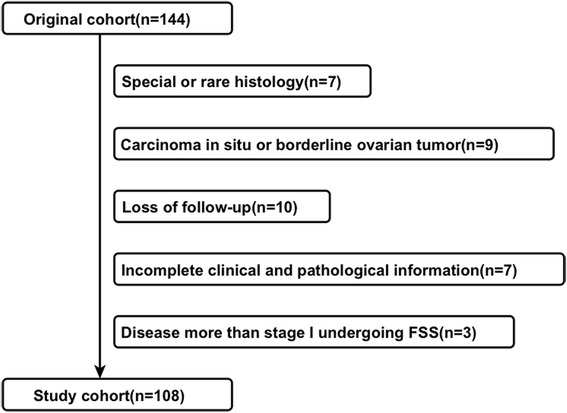
Patient enrollment

A review of the database revealed 144 patients with stage I EOC aged ≤ 40 years at diagnosis during the study period (1999–2013). Overall, 108 patients met the inclusion criteria and were included in the analysis. The selection process and reasons for patient exclusion are summarized in Fig. 1. Eleven patients with high-risk disease wanted to preserve their fertility and underwent FSS. Altogether, 52 (48.1%) patients underwent FSS and 56 (51.9%), RS.

### Comparison of clinical and pathological features

Clinical and pathological variables are shown in Table 1. The median age of patients at diagnosis who underwent FSS was significantly younger (by 10 years), compared with those who underwent RS (*p* < 0.001). Significantly more patients who underwent FSS (94.2%) were nulliparous, compared with those underwent RS (32.1%; *p* < 0.001). Median tumor size in the FSS group was significantly larger than that in the RS group (*p* = 0.002); this could be due to the significantly higher proportion of mucinous histology (*p* < 0.001) in the FSS group. Most of the tumors were well-differentiated (66.7%). Eight patients had mixed tumor types. For easy statistical analysis, the epithelial component which took the larger proportion of the tumor was considered as the predominant histology. Because the RS group included more high-grade and clear-cell tumors (*p* < 0.001) than the FSS group, the proportion of patients receiving adjuvant chemotherapy (*p* = 0.006) were higher in the RS group. Patients in the RS group were more likely to have coexisting endometriosis (*p* = 0.006), which could be attributed to the higher proportion of clear-cell histology.

**Table 1:** Clinical and pathological features of stage I EOC patients of reproductive age

Variables	All (*N* = 108)	RS (*N* = 56; 51.9%)	FSS (*N* = 52; 48.1%)	*P* value
Age at diagnosis(years) Median(min–max)	30(16–40)	35(17–40)	25(16–40)	< 0.001
≤ 30	55(50.9%)	10(17.9%)	45(86.5%)	< 0.001
> 30	53(49.1%)	46(82.1%)	7(13.5%)	
Nulliparous—*N*(%)	67(62.0%)	18(32.1%)	49(94.2%)	< 0.001
Primary tumor size(cm) Median(min–max)	12.0(3.0–40.0)	10.0(3.0–28.0)	15.0(4.0–40.0)	0.002
Side of ovarian tumor—*N*(%)				0.087
Both	5(4.6%)	5(8.9%)	0(0%)	
Left	56(51.9%)	28(50.0%)	28(53.8%)	
Right	47(43.5%)	23(41.1%)	24(46.2%)	
Pretreatment tumor markers—*N*(%)				0.084
Normal	55(50.9%)	33(58.9%)	22(42.3%)	
Elevated	53(49.1%)	23(41.1%)	30(57.7%)	
Histology—*N*(%)				< 0.001
Mucinous	52(48.2%)	14(25.0%)	38(73.1%)	
Serous	9(8.3%)	8(14.3%)	1(1.9%)	
Endometrioid	27(25.0%)	16(28.6%)	11(21.2%)	
CCC	20(18.5%)	18(32.1%)	2(3.8%)	
Grade—*N*(%)				< 0.001
G1	72(66.7%)	27(48.2%)	45(86.5%)	
G2	12(11.1%)	8(14.3%)	4(7.7%)	
G3	4(3.7%)	3(5.4%)	1(1.9%)	
CCC	20(18.5%)	18(32.1%)	2(3.9%)	
FIGO stage—*N*(%)				0.633
IA–B	36(33.3%)	17(30.4%)	19(36.5%)	
IC1	55(50.9%)	31(55.3%)	24(46.2%)	
IC2–3	17(15.7%)	8(14.3%)	9(17.3%)	
Approach of staging—*N*(%)				0.932
LPS	5(4.6%)	3(5.4%)	2(3.8%)	
LPT	103(95.4%)	53(94.6%)	50(96.2%)	
Surgical staging procedures—*N*(%)				
Omentectomy	104(96.3%)	56(100%)	48(92.3%)	0.109
Appendectomy	104(96.3%)	56(100%)	48(92.3%)	0.109
Lymphadenectomy	103(95.4%)	55(98.2%)	48(92.3%)	0.317
Para-aortic LN excision	58 (53.7%)	34(60.7%)	24(46.2%)	0.186
Number of LN-median(min–max)	18(2–57)	17(5–53)	22(2–57)	0.215
Chemotherapy—*N*(%)				0.006
Taxane platinum	52(48.1%)	31(55.4%)	21(40.4%)	
Platinum-based regimen	31(28.7%)	19(33.9%)	12(23.1%)	
No chemotherapy	25(23.2%)	6(10.7%)	19(36.5%)	
Cycles-median(min–max)	4(3–9)	6(3–9)	3(3–9)	0.001
EAOC—*N*(%)	21(19.4%)	17(30.4%)	4(7.7%)	0.006
EC—*N*(%)	4(3.7%)	4(7.1%)	0(0%)	0.146
Follow–up (months) Median(min–max)	83(9–216)	96(9–216)	60(34–209)	0.026

*RS* radical surgery, *FSS* fertility-sparing surgery, *LPS* laparoscopy, *LPT* laparotomy, *EOC* epithelial ovarian cancer, *CCC* clear-cell carcinoma, *EAOC* endometriosis-associated ovarian cancer, *EC* endometrial carcinoma

### Comparison of oncologic outcomes

After a median follow-up of 83 months (range, 9–216 months), 14 (13.0%) patients relapsed, 8 (7.4%) died of progressive disease, and 100 (92.6%) were censored in the entire study cohort. The 5-year TSS and DFS rates were 92.6 and 86.6%, respectively. RS and FSS patients had a 5-year TSS rate of 89.3 and 97.3%, respectively, and a 5-year DFS rate of 83.0 and 91.0%, respectively.

**Fig. 2:**
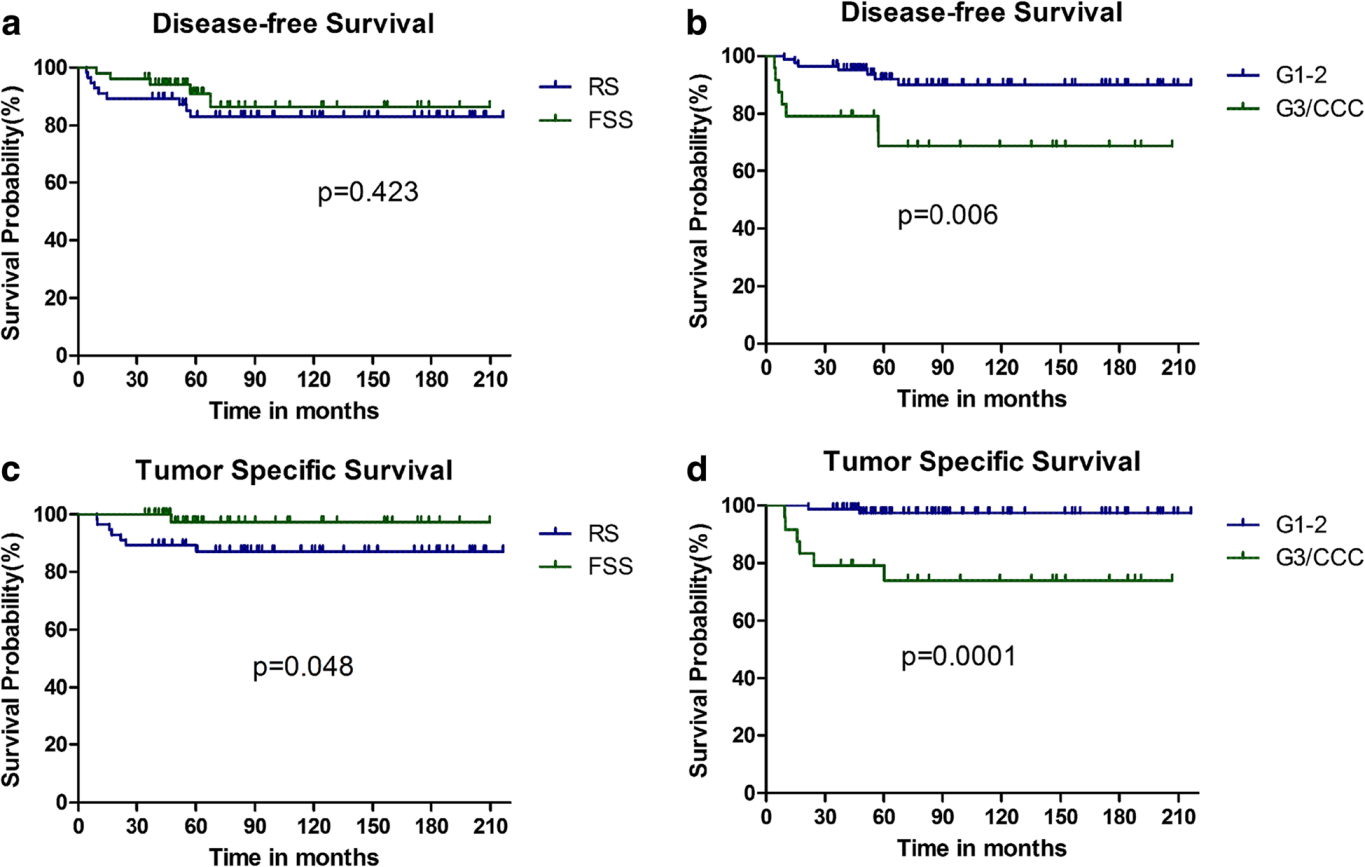
Comparison of survival in women of reproductive age with stage I epithelial ovarian cancer. Kaplan-Meier survival curves showing the effect of FSS or RS on disease-free survival (**a**) (*p* = 0.423) and tumor-specific survival (**c**) (*p* = 0.048); the effects of grade 1-2 or grade 3/clear-cell carcinoma on disease-free survival (**b**) (*p* = 0.006) and tumor-specific survival (**d**) (*p* = 0.0001)

Tables 2 and 3 show the results of the univariate and multivariate survival analyses of DFS and TSS, respectively. Surgery type, age, tumor size, histology, tumor grade, FIGO sub-stage, pretreatment tumor markers, and EAOC were included in the univariate analysis. Patients with grade 1-2 tumor tended to have higher 5-year DFS (*p* = 0.006) and TSS (*p* < 0.001) rates than those with a grade 3 or CCC; grade 3 and CCC were considered together (Fig. 2b–d). FSS patients tended to have better TSS than RS patients (*p* = 0.048). However, this was not observed with DFS (*p* = 0.423) (Fig. 2a–c). Women aged ≤ 30 years also had better TSS (*p* = 0.030) but not DFS (*p* = 0.106), than those aged > 30 years. Patients with EAOC had a worse DFS (*p* = 0.002) and TSS (*p* = 0.024) than those without EAOC. No adverse effects of FIGO stage IC1 or IC2-3 versus IA-B on prognosis were observed (DFS, *p* = 0.053; TSS, *p* = 0.314).

**Table 2:** Risk factors related to DFS in stage I EOC patients of reproductive age

Variables	Relapse		5 year-DFS%	*p* value^a^	*p* value^b^	HR(95%CI)
	Yes	No				
Type of surgery				0.423		
RS (reference)	9	47	83.0			
FSS	5	47	91.0			
Age				0.106		
≤ 30 years (reference)	4	51	91.7			
> 30 years	10	43	81.9			
Tumor size				0.878		
≤ 12 cm (reference)	7	48	88.0			
> 12 cm	7	46	85.2			
Histology				0.467		
Mucinous	4	48	91.5			
Serous	1	8	88.9			
Endometrioid	5	22	83.1			
CCC (reference)	4	16	78.5			
Grade				0.006	0.048	3.41(1.01–11.49)
G1-2 (reference)	7	77	92.0			
G3/CCC	7	17	68.6			
FIGO stage				0.053		
IA-B (reference)	1	35	97.2			
IC1	9	46	83.9		0.199	4.03(0.48–33.87)
IC2-3	4	13	70.6		0.325	3.35(0.30–37.41)
Pretreatment tumor markers				0.071	0.075	3.30(0.89–12.29)
Normal (reference)	4	51	92.2			
Elevated	10	43	80.9			
EAOC				0.002	0.092	2.56(0.86–7.63)
No (reference)	7	80	91.5			
Yes	7	14	67.3			

*RS* radical surgery, *FSS* fertility-sparing surgery, *EOC* epithelial ovarian cancer, *CCC* clear-cell carcinoma, *EAOC* endometriosis-associated ovarian cancer, *DFS* disease-free survival, *HR*: hazard ratios, *CI* confidence intervals

^a^Log-rank test

^b^Cox proportional hazards model

**Table 3:** Risk factors related to TSS in stage I EOC patients of reproductive age

Variables	DOD		5 year-OS%	*p* value^a^	*p* value^b^	HR(95%CI)
	Yes	No				
Type of surgery				0.048	0.469	0.38(0.03–5.34)
RS (reference)	7	49	87.2			
FSS	1	51	97.3			
Age				0.030	0.690	1.81(0.10–33.40)
≤ 30 years (reference)	1	54	97.6			
> 30 years	7	46	86.3			
Tumor size				0.495		
≤ 12 cm (reference)	5	50	90.1			
> 12 cm	3	50	93.9			
Histology				0.074		
Mucinous	1	51	97.5		0.195	11.38(0.29–450.86)
Serous	1	8	88.9		0.235	4.46(0.38–52.35)
Endometrioid	2	25	92.6		0.078	7.80(0.80–76.36)
CCC (reference)	4	16	78.5			
Grade				< 0.001	0.007	38.92(2.68–565.45)
G1-2 (reference)	2	82	97.3			
G3/CCC	6	18	73.9			
FIGO stage				0.314		
IA-B (reference)	1	35	97.2			
IC1	6	49	88.6			
IC2-3	1	16	90.0			
Pretreatment tumor markers				0.430		
Normal (reference)	3	52	94.5			
Elevated	5	48	89.0			
EAOC				0.024	0.343	2.34(0.40–13.59)
No (reference)	4	83	95.1			
Yes	4	17	79.6			

*RS* radical surgery, *FSS* fertility-sparing surgery, *TSS* tumor-specific survival, *EOC* epithelial ovarian cancer, *CCC* clear-cell carcinoma, *EAOC* endometriosis-associated ovarian cancer, *DOD* dead of the recorded disease, *HR* hazard ratios, *CI* confidence intervals

^a^Log-rank test

^b^Cox proportional hazards model

We included tumor grade (*p* = 0.006), FIGO stage (*p* = 0.053), EAOC (*p* = 0.002), and pretreatment tumor markers (*p* = 0.071) in the multivariate analysis of DFS. Multivariate analysis confirmed the high risk for tumor grade (grade 3/CCC vs grade 1-2; HR 3.41, 95% CI 1.01–11.49, *p* = 0.048) for DFS. Moreover, the type of surgery (*p* = 0.048), age (*p* = 0.030), histology (*p* = 0.074), tumor grade (*p* < 0.001), and EAOC (*p* = 0.024) were included in the multivariate analysis of TSS. Multivariate analysis also confirmed the high risk for tumor grade (grade 3/CCC vs grade 1-2; HR 38.92, 95% CI 2.68-565.45, *p* = 0.007) in TSS.

### Subgroup analysis based on high-risk and low-risk disease

Overall, 38 (35.2%) patients had high-risk disease (grade 3 or stage IC2-3 or clear-cell carcinoma). Among them, 11 (28.9%) patients underwent FSS and 27 (71.1%) patients, RS.

**Fig. 3:**
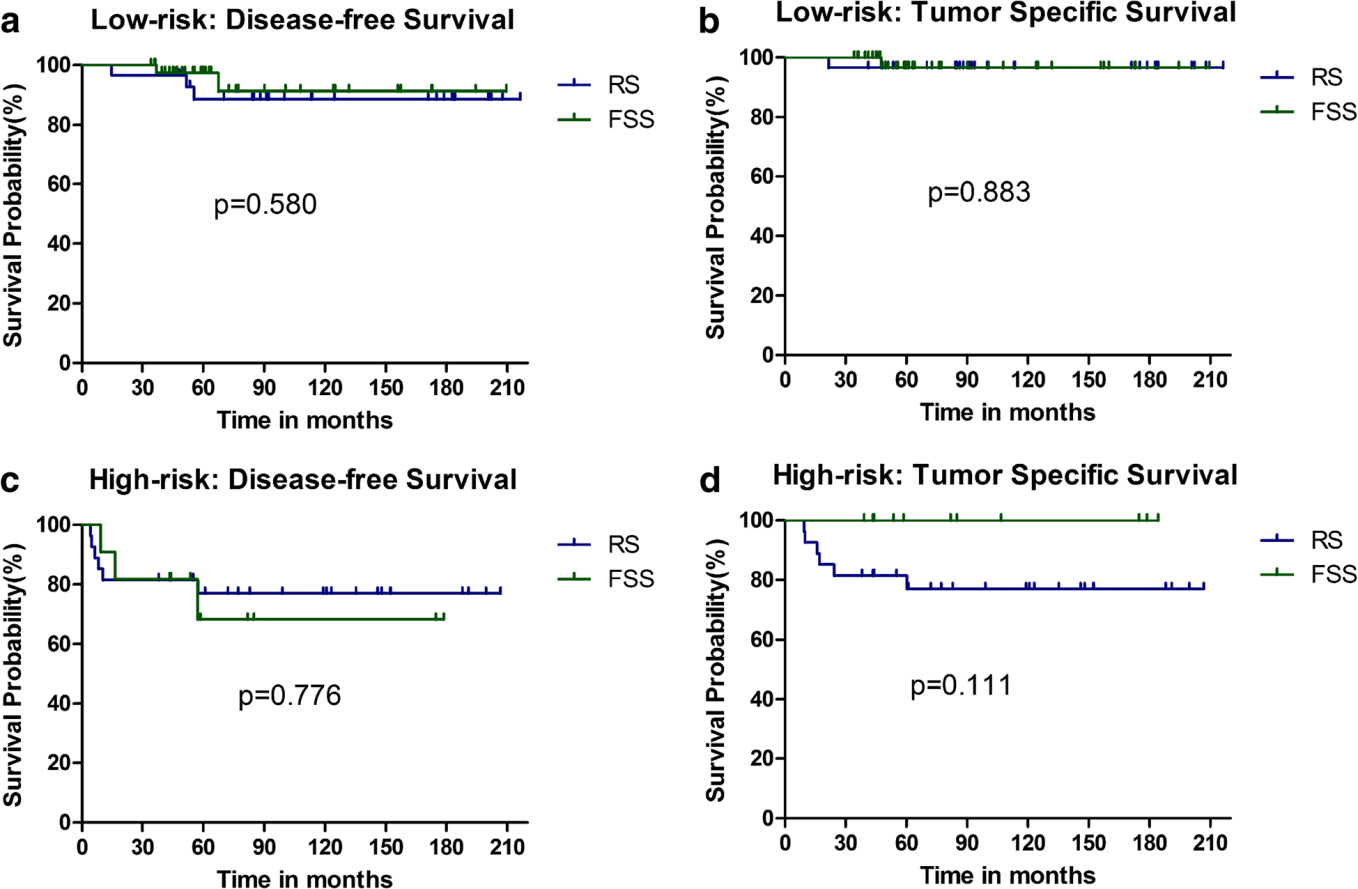
Comparison of survival in patients with low- and high-risk stage I epithelial ovarian cancer. Kaplan-Meier survival curves showing the effects of FSS or RS on disease-free survival (**a**) (*p* = 0.580) and tumor-specific survival (**b**) (*p* = 0.883) among low-risk patients; the effect of FSS or RS on disease-free survival (**c**) (*p* = 0.776) and tumor-specific survival (**d**) (*p* = 0.111) among high-risk patients

Among the low-risk patients, those who underwent RS and FSS had a 5-year DFS rate of 88.7 and 91.3%, a 5-year TSS rate of 96.6 and 96.6%, with no statistical significance (DFS, *p* = 0.580; TSS, *p* = 0.883) (Fig. 3a–b). The high-risk RS and FSS patients had a 5-year DFS rate of 77.0 and 68.2, a 5-year TSS rate of 77.0 and 100%, and also with no statistical significance (DFS, *p* = 0.776; TSS, *p* = 0.111) (Fig. 3c, d).

### The distribution of grade 3 and clear-cell carcinoma in stage I EOC cohort of reproductive age

Since grade 3 and clear-cell carcinoma was the only independently significant predictor of DFS and TSS, we evaluated the correlation of grade 3 and clear-cell carcinoma with age, endometriosis, and surgery type. As can be seen from Table 4, 87.5% of patients with grade 3/CCC versus 38.1% of patients with grade 1-2 aged more than 30 years (*p* < 0.001); 41.7% of patients with grade 3/CCC versus 13.1% of patients with grade 1-2 were coexistent with endometriosis (*p* = 0.002); and 87.5% of patients with grade 3/CCC versus 41.7% of patients with grade 1-2 underwent radical surgery (*p* < 0.001).

**Table 4:** The correlation of grade 3/CCC with age, endometriosis, and FSS

Variables	Grade1-2 (*N* = 84)	Grade 3/CCC (*N* = 24)	*p* value
Age			< 0.001
≤ 30	52(61.9%)(94.5%)	3(12.5%)(5.5%)	
> 30	32(38.1%)(60.4%)	21(87.5%)(39.6%)	
Endometriosis			0.002
No	73(86.9%)(83.9%)	14(58.3%)(16.1%)	
Yes	11(13.1%)(52.4%)	10(41.7%)(47.6%)	
Type of surgery			< 0.001
RS	35(41.7%)(62.5%)	21(87.5%)(37.5%)	
FSS	49(58.3%)(94.2%)	3(12.5%)(5.8%)	

We calculated both the row and column percents. The first percent in the cell was the column percent representing a proportion in the “grade” arm, and the second was the row percent representing a proportion in the “age,” “endometriosis,” and “FSS or RS” arm

*CCC* clear-cell carcinoma, *RS* radical surgery, *FSS* fertility-sparing surgery

### Comparison of pattern of recurrence

As can be seen from Table 5, 22.2% (2/9) in the RS group versus 80.0% (4/5) in FSS had localized relapses. In the FSS group, 80.0% of recurrences were confined to the contralateral ovary. Furthermore, 71.4% (5/7) in grade 1-2 versus 14.3% (1/7) in grade 3/CCC had localized relapses.

**Table 5:** Pattern of recurrence and oncologic outcomes of the relapsed patients

Arm	Age	Histology, grade, stage	RFI (M)	Relapse sites	Relapse pattern	Salvage treatment	Outcomes
RS.1	34	Endometrioid, G3, IC1	8.1	Vaginal stump, liver, bladder, diaphragm, ileum, ascending colon	Disseminated	RCRS + chemotherapy	DOD
RS.2	36	CCC, IA	10.3	Liver, pelvic mass	Disseminated	Chemotherapy	DOD
RS.3	37	Endometrioid, G1, IC1	14.6	Vaginal stump, rectal mass	Localized	Chemotherapy	DOD
RS.4	40	Mucinous,G1,IC1	51.7	Lung	Disseminated	Chemotherapy	AWD
RS.5	36	Endometrioid, G1, IC1	55.3	Vaginal stump, ileum, para-urethra	Localized	RCRS + chemotherapy + radiotherapy	AWD
RS.6	34	CCC, IC1	4.9	Systematic lymph nodes	Disseminated	Chemotherapy + radiotherapy	DOD
RS.7	40	CCC, IC3	57.3	Abdominopelvic cavity	Disseminated	Chemotherapy	DOD
RS.8	31	HGSC, IC1	4.2	Abdominopelvic cavity	Disseminated	Chemotherapy	DOD
RS.9	36	CCC, IC1	6.4	Abdominopelvic cavity	Disseminated	Palliative	DOD
FSS.1	24	Endometrioid, G3, IC3	57.1	Contralateral ovary	Localized	RCRS + chemotherapy	NED
FSS.2	34	Endometrioid, G1, IC1	67.4	Contralateral ovary	Localized	RCRS + chemotherapy	NED
FSS.3	29	Mucinous, G1, IC2	9.2	Contralateral ovary	Localized	RCRS + chemotherapy	AWD
FSS.4	22	Mucinous, G1, IC1	36.9	Lung	Disseminated	Chemotherapy + radiotherapy	DOD
FSS.5	25	Mucinous, G1, IC3	16.3	Contralateral ovary	Localized	RCRS + chemotherapy	NED

*RS* radical surgery, *FSS* fertility-sparing surgery, *CCC* clear-cell carcinoma, *RFI* relapse-free intervals, *DOD* dead of the recorded disease, *NED* no evidence of disease, *AWD* alive with the recorded disease, *RCRS* re-cytoreductive surgery, *HGSC* high-grade serous carcinoma, *TSS* tumor-specific survival

Four in five FSS patients had a localized relapse in the contralateral ovary; the remaining patient had a disseminated relapse in the lung. Most patients in the RS group had multiple relapse sites and lost the opportunity to undergo follow-up surgery.

Taking a closer look at outcomes, seven (77.8%) of nine patients in the RS group had relapses and one (20.0%) of five patients in the FSS group who relapsed died of progressive disease; the remaining two (22.2%) of nine patients in the RS group and one (20.0%) of five patients in the FSS group were alive with the disease; another three (60.0%) patients in the FSS group were salvaged with repeated surgery and long-term survival without tumor was achieved.

### Reproductive outcomes for patients in the FSS group

Of 52 patients in the FSS group, 34 (65.4%) attempted to become pregnant. Five (14.7%) patients were unable to conceive and diagnosed with infertility. Among the remaining 29 patients, 32 pregnancies were recorded, including 28 live births (82.4%, 28/34), 1 induced abortion, 2 miscarriages, and 1 intrauterine death. None of the patients underwent radical surgery after child-bearing.

Two patients failed to become pregnant and had recurrent disease of the retained ovary, concurrent with unexpected endometrial malignancy. One patient in the FSS group had an endometriosis relapse rather than malignant tumor on the contralateral ovary before she successfully became pregnant; the contralateral ovary was then resected. In another patient, relapse occurred in the contralateral ovary at 32 weeks of gestation. A cesarean section and restaging surgery were performed; the child was delivered and the woman survived without tumor for a long time.

## Discussion

In this study, we evaluated women of reproductive age with stage I EOC who underwent FSS or RS. Grade 3/CCC was the only significant independent risk factor for DFS and TSS. Tumor-specific survival in FSS was better than that in the RS group in univariate analysis, because up to 71.1% of the high-risk patients underwent RS. In addition, up to 87.5% of patients with grade 3/CCC underwent RS. In the cohort, we defined the tumor with stage IC2-3 or grade 3 or clear-cell histology as high-risk disease. Several studies have already confirmed that no significant difference in patient survival exists between stage IC1 and stage IA tumors [10, 11]. Based on these data, we did not classify the tumor stage IC1 as high-risk disease, which is different from previous studies [12, 13]. The survival advantage of FSS compared with RS was not observed among high-risk patients. Similar to our study, studies published to date comparing FSS and RS have found no significant influence of FSS on prognosis, even among high-risk patients, including those with stage IC1-3, grade 3, or clear-cell carcinoma [12, 13]. Grade 3 and CCC were considered together in our cohort, because there were only four grade 3 tumors. This was understandable because malignant ovarian tumors confined to stage I were usually well-differentiated [13].

In an Italian study, researchers evaluated 240 patients with early-staged EOC (eEOC) treated with FSS [14]. Similar to our study, they found that grade 3 tumors were the only factor that negatively affected the prognosis of patients [14]. They subsequently evaluated 1031 patients with eEOC treated with FSS or RS [13] and found that grade 3 tumor was associated with shorter DFS and overall survival. However, in both studies, the classification of the tumor differentiation of CCC was not specified [13]. Although we did not observe the effect of sub-staging on DFS in our study, the *p* value (0.053) indicated a little significance. Most studies reported adverse effect of stage IC2/3 on DFS [10, 12, 13].

Our preliminary study has found that patients with EAOC might own an improved survival, but endometriosis per se was not an independently significant predictor in the multivariate analysis [9, 15, 16]. However, focusing on young (aged ≤ 40 years) patients with stage I EOC, EAOC patients had significantly poorer DFS and TSS than those with non-EAOC in the univariate analysis. It is possible that this trend is due to a higher proportion of grade 3/CCC in patients with EAOC than in those with non-EAOC (47.6 vs. 16.1%, *p* = 0.002).

Similarly, patients aged ≤ 30 years had a better TSS than those aged > 30 years in the univariate analysis. The incidence of grade 3/CCC was significantly greater in patients after they were aged 30 years. Patients aged > 30 years had significantly more grade 3/CCC than those aged ≤ 30 years (39.6 vs. 5.5%, *p* < 0.001). The literature suggests that younger age is more correlated with low-grade tumors [13], and increased age, with worse overall survival [12]. Patients with stage I EOC at a reproductive age tended to have a mucinous histology, low-grade, sub-staged early, and had better intrinsic biological behavior, compared with those at a non-reproductive age [13]. Therefore, the high-risk of age > 30, endometriosis, and radical surgery on survival in univariate analysis was attributed to the higher proportion of grade 3/CCC.

It should also be noted that the preservation of the uterus and contralateral ovary does not seem to affect patient survival [17]. In a Japanese study, researchers evaluated 16 patients with stage I CCC who underwent FSS, 205 patients who underwent RS, and 64 patients with stage I non-CCC who underwent FSS [18]. They found that patients with stage I CCC who underwent FSS did not have a poorer prognosis than those receiving RS and those with non-CCC who underwent FSS [18]. Researchers suggested that FSS was adequate for patients with stage I EOC, regardless of the stage, grade, and histological subtype [13].

In this study, the pattern of recurrence was more advantageous in FSS than that in the RS group. According to literature, relapse on the retained ovary has a good possibility of rescue with surgery and chemotherapy and did not affect the long-term survival of FSS patients [7, 19, 20]. Whereas patients who relapse in the lymph nodes and widely spread in the peritoneum, which is typical of clear cell histology, had a poor prognosis [4]. Compared with grade 1-2 tumors, grade 3 tumors give rise to a higher rate of extra-ovarian recurrences [14, 20]. Thus, the higher proportion of an isolated ovarian recurrence after FSS in our study could also be due to the lower proportion of grade 3/CCC. In our study, we found no evidence of fertility damage among patients in the FSS group. Previously published data suggest that there is an 80% rate of successful pregnancy after FSS [7, 14]. Besides, more fertility-preservation techniques, such as ovarian tissue cryopreservation and pharmacological protection against gonadotoxic agents, are developed to prevent the loss of reproductive fitness in these women [21, 22].

Considering the unbalanced distribution of grade 3/clear-cell carcinoma between groups, only 12.5% (3/24) of patients with grade 3 or clear-cell carcinomas underwent FSS, and the safety of FSS for patients with grade 3 or clear-cell carcinomas was uncertain. Patients aged ≤ 30 had only three (5.5%, 3/55) grade 3/clear-cell carcinomas, reflecting that young women tended to have more well-differentiated tumors. In addition, 86.5% (45/52) of patients in the FSS group were aged ≤ 30, indicating that FSS was safe in this patient population.

## Conclusion

Grade 3 or clear-cell carcinoma was the only independent risk factor for survival of patients of reproductive age with stage I EOC. FSS can be safely performed on patients of reproductive age with grade 1-2, stage I EOC. The safety of FSS for grade 3 and clear-cell carcinoma warrants further investigation.

## Abbreviations

CCC: Clear-cell carcinoma
DFS: Disease-free survival
EAOC: Endometriosis-associated ovarian cancer
EOC: Epithelial ovarian cancer
FSS: Fertility-sparing surgery
RS: Radical surgery
TSS: Tumor-specific survival
